# New solution for old challenge: Device closure of non-centrally positioned atrial septal defect

**DOI:** 10.34172/jcvtr.2020.40

**Published:** 2020-08-04

**Authors:** Shamsi Ghaffari, Akbar Molaei, Ahmad Jamei Khosroshahi, Rezvaniyeh Salehi, Mehrnoush Toufan Tabrizi, Mahmood Samadi

**Affiliations:** ^1^Cardiovascular Research Center, Tabriz University of Medical Sciences, Tabriz, Iran; ^2^Cardiovascular Research Center, Faculty of Medicine, Tabriz University of Medical Sciences, Tabriz, Iran

**Keywords:** Multiple ASDs, Peripheral ASD, Transcatheter Closure

## Abstract

Multiple interatrial defects, termed fenestrated ASDs that require closure are not uncommon. The problem arises when a centrally located defect or a patent foramen oval (PFO) is associated with another peripherally located defect. In cases like this, all attempts at crossing the true defect might totally fail or might be difficult because the wire or the catheter crosses the central defect repeatedly despite the use of a sizing balloon. In order to overcome such an issue, we introduce a new technique by which not only the procedure and the fluoroscopy time will be reduced, but also it ceases the mistakes about the number of defects, their size and location.

## Introduction

Multiple atrial septal defects (ASDs) are approximately detected in 10% of patients with ASDs. Centrally located, secundum defects are ideal for device closure, but there is considerable variation in size and location of the defects. A small proportion of ASDs may have multiple fenestrations and these are often considered unsuitable for device closure.^1^ A decent image of the defect is crucial in determining accurate measurement of the defect and subsequently in selecting the appropriately sized device. The use of two- and three-dimensional transesophageal (TEE)^1,2^ or intracardiac echocardiography (ICE)^3^ provided useful information for transcatheter closure of multiple ASDs. Cardiac computerized tomography (CT) is more useful than a 2D echocardiogram in adults. The use of a sizing balloon in selecting the appropriately sized device is controversial.

Patients with multiple defects can be effectively treated with Transcatheter techniques, including single device closure, multiple devices in one procedure and multiple devices in staged procedures, and with surgical repair.^4,5^ The problem arises when a centrally located defect or a patent foramen oval (PFO) is associated with another defect that is located peripherally.

In cases like this, every attempt at crossing the true defect might totally fail because the wire or catheter crosses the central defect repeatedly despite the use of a sizing balloon.^4,6^

In order to overcome such an issue, we introduce a new technique to ease crossing the true or peripherally located defect. This procedure facilitates crossing the true or peripherally located defect. We report two cases that we encountered this problem.

## Case Presentation

### Case 1

**Figure 1:**
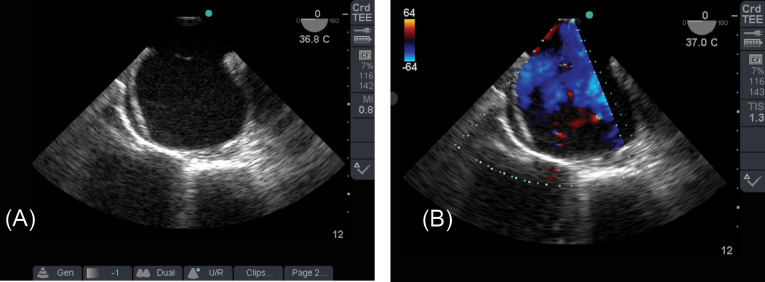
TEE 2D image shows two large defects of interatrial septum (A). TEE color image shows left to right shunt via defects (B)

A 38-year old woman weighing 65 kg with two large ASDs (Figure 1A and B).

**Figure 2:**
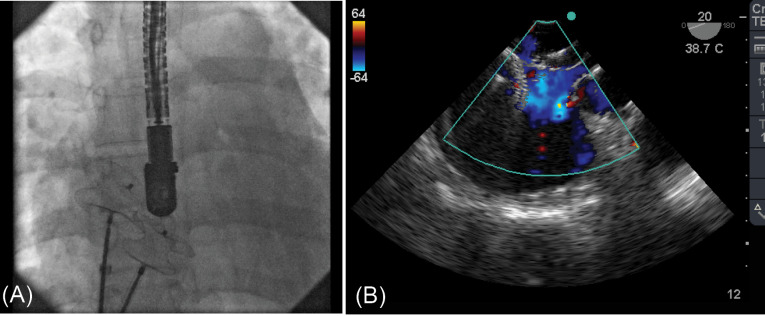
Fluoroscopy image shows interleaved two devices to each other (A). TEE color image shows significant residual shunt via atrial septum (B)

During transcatheter closure of the defect, both guide wires and long sheaths were crossing the central defect repeatedly despite closing this defect by a sizing balloon (Figure 2A and B).

The patient was referred for a surgical closure since we supposed there was an extra (third) defect, but in the operation room the surgeon declared that they were only two. Post-operative follow-up was uneventful with no evidence of residual shunt.

### Case 2

**Figure 3:**
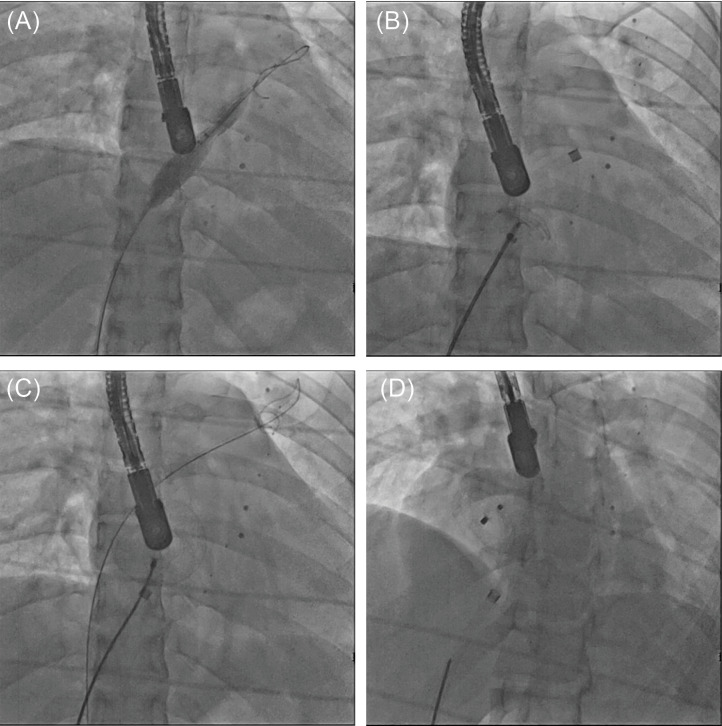
Fluoroscopy image shows guidewire and sizing balloon crossed via PFO. (B) Fluoroscopy image shows PFO closure by device and cross the true defect by long sheath. (C) The fluoroscopy image shows deployed ASD occluder after recapture of PFO device while preserving of the exchange wire in place. (D) The fluoroscopy image shows released ASD device after removal of the exchange wire

A 27-year old woman weighing 56 kg with peripheral large ASD secundum accompanied by a large PFO. All attempts at crossing the large defect were unsuccessful because the PFO got in the way annoyingly even after its closure by a sizing balloon (Figure 3A).

Therefore, we decided to close the PFO by a device temporarily (Figure 3B).

Then after that, we could cross the true ASD conveniently. Before deployment of the second device, we recaptured the first device but kept the exchange wire in place. After deploying the second device, we checked the status and position of the device (Figure 3C).

The device had covered the PFO, so the exchange wire was extracted and then the device was released in place with good position without residue (Figure 3D).

## Discussion

Multiple or fenestrated ASDs that require closure are not uncommon,^7,8^ and the approaches to Transcatheter closure of multiple ASDs are not in harmony with each other.

Moreover, there is considerable morphological variation in size and location of the defects. Therefore, there are different approaches to percutaneous closure.^4,8^ There are some unsuccessful reports about percutaneous approach and surgical refer of these patients due to the considerable residual shunt after device closure.^4,8^

The problem arises when a centrally located defect or a PFO is associated with another peripherally located defect. The use of TTE, TEE or ICE accompanied by fluoroscopy facilitates the procedure and crossing the defect.^1,3^ In cases like this, the attempt at crossing the true defect might totally fail or might be difficult because the wire or catheter crosses the central defect repeatedly despite the use of a sizing balloon.^4-6^

We encountered this particular problem in patients as mentioned above despite closing the central defect by a sizing balloon. As far as we can gather, this problem could be the main cause of the residual shunt in previous unsuccessful reports. Here in, the operator may think of an extra (A third) defect erroneously, like what we did in case 1, and refer the patient for surgery or impose an additional device or procedure on the patient.

Accordingly, what we recommend is closing the PFO or central defect by a device temporarily and then crossing the true or peripheral ASD conveniently.

Before deployment of the second device, we should recapture the first device (if necessary) while the exchange wire is kept in place. After deployment but before releasing the second device, we check the status and position of the device and the other defect for double device closure. If there is not any additional defect or significant residual shunt, it is recommended to remove the exchange wire and release the second device. This procedure facilitates crossing the true or peripherally located defect.

By reviewing the literature, we find this to be a novel technique; by means of which we succeed in overcoming the above-mentioned long-standing problem.

This newly introduced technique, not only helps to decrease the procedure and fluoroscopy time, but also it ceases the mistakes about the number of defects, their size and location.

## Competing interests

None declared.

## Ethical approval

Informed consent was obtained from the patients for publiction of the case.

## Funding

None.
